# Thyroid Dysfunction after Gonadotropin-Releasing Hormone Agonist Administration in Women with Thyroid Autoimmunity

**DOI:** 10.1155/2022/6331657

**Published:** 2022-04-12

**Authors:** Loris Marin, Guido Ambrosini, Marco Noventa, Flavia Filippi, Eugenio Ragazzi, Francesco Dessole, Giampiero Capobianco, Alessandra Andrisani

**Affiliations:** ^1^Department of Women's and Children's Health, University of Padua, Padua 35100, Italy; ^2^Department of Pharmaceutical and Pharmacological Sciences, University of Padua, Padua 35100, Italy; ^3^Department of Surgical, Microsurgical and Medical Sciences, Gynecologic and Obstetric Clinic, University of Sassari, Sassari 07100, Italy

## Abstract

GnRH agonists (GnRHa) are a useful tool for pretreatment before artificial endometrial preparation for frozen-thawed embryo-transfer (FET). Their prolonged administration has been associated with thyroid dysfunction, both hyper and hypothyroidism. The aim of this study is to investigate the impact of GnRHa administration on thyroid function in women undergoing artificial endometrial preparation. Seventy-eight euthyroid women undergoing endometrial preparation with hormone replacement for FET were retrospectively reviewed. They were divided into two groups according to pretreatment with GnRHa (group A, 42 women) or with an oral contraceptive (group B, 36 women). Group A was subsequently divided into two subgroups according to thyroid autoimmunity presence. Thyroid function has been evaluated and compared among groups and subgroups. Our results did not show any statistically significant differences in age, body mass index, and basal thyroid stimulating hormone (TSH). Total estradiol dosage, duration of treatment, and endometrial thickness were comparable among groups. When TSH was measured 14 days after embryo transfer, no significant differences between the two groups were reported. Among women of group A, TSH was significantly higher only in women with thyroid autoimmunity. GnRHa seems to be associated with thyroid dysfunction in women with thyroid autoimmunity undergoing hormone replacement therapy for FET.

## 1. Introduction

Thyroid function has a crucial role in fertility and pregnancy outcomes [1] and may have a role in assisted reproductive technologies (ARTs) success rates since it has been associated with adverse outcomes [2, 3]. Nevertheless, treatments concerning ART can cause changes in the hypothalamic-pituitary-thyroid axis, mainly through high circulating sex hormone levels and changes in thyroid hormone binding proteins [4].

Gonadotropin-releasing hormone agonist (GnRHa) is a drug widely used in the gynecological field to treat estrogen-dependent diseases. After an initial flare-up effect with massive liberation of gonadotropins, it causes gonadotropin-releasing hormone (GnRH) receptor downregulation and consequent rapid hypoestrogenism. For its mechanism of action, the LH surge is not possible and spontaneous ovulation is prevented [5]. In ARTs it has been used both during controlled ovarian hyperstimulation cycles [6] and in artificial frozen-thawed embryo transfer cycles [7–9].

In the scientific literature the usage of GnRHa in depot formulation has been associated with thyroid dysfunctions, both with hyper and hypothyroidism [4, 10–13]. The data about this topic are few and of limited quality, as there are only retrospective data [4, 10, 11, 13]. For this reason, the mechanism of action of GnRHa on thyroid function is not clear. The aim of our study was to study the impact of GnRHa on thyroid function in women undergoing frozen-thawed embryo transfer cycles.

## 2. Materials and Methods

### 2.1. Patients

We retrospectively evaluated 78 infertile women undergoing one frozen-thawed embryo transfer from May 2018 to February 2020 at our IVF unit (Department of Women's and Children's Health, University of Padua, Italy). Inclusion criteria were as follows: age <45 years, body mass index (BMI) between 18 and 30 kg/m^2^, no uterine pathologies, and artificial endometrial preparation. As per protocol, antithyroid peroxidase (TPO) and/or antithyroglobulin (TG) antibodies are evaluated in all infertile patients in our center. Patients were divided into two groups according to pretreatment protocol: GnRHa (Decapeptyl® 3.75 mg/2 ml, Ipsen) (group A) or OCP (oral contraceptive pill: dienogest 2 mg/ethinyl estradiol 0.03 mg, Sibilla®, Gedeon Richter) (group B). Both pretreatment protocols before FET are decided indifferently by the clinician. Women in group A received GnRHa depot administration during the midluteal phase of menstrual cycle. Two days after the next menstrual period, they started receiving hormonal replacement therapy. Instead, women in group B received OCP for 14–21 days, and two days after discontinuation, they started receiving hormonal replacement therapy. Group A was subsequently divided into two subgroups (A1 and A2) according to the presence or absence of at least one of the evaluated thyroid antibodies. Written informed consent was obtained from all participants, and the study was conducted as per the Declaration of Helsinki. This study was approved by the local ethics committee with protocol *n*. 0065680.

### 2.2. Artificial FET Protocols

Two days after the menstrual period following GnRHa administration or two days after OCP discontinuation, oral estradiol (Progynova® 2 mg, Bayer) was administered three times a day. After eight or nine days, endometrial thickness, defined as the transvaginal ultrasonography measurement of endometrium at the maximal distance between each myometrial/endometrial interface, was evaluated. If endometrium thickness was <7 mm, estradiol dose was increased up and endometrium thickness was re-evaluated after 4–5 days. When endometrium thickness was ≥7 mm, the woman was instructed to continue with the same dosage of estradiol and to start with vaginal progesterone tablets (Progeffik®, 200 mg, Effik Italia) three times a day. The therapy was continued for 14 days after the embryo transfer, until the serum beta-hCG test.

### 2.3. Laboratory Assays

Serum thyroid autoantibodies were measured in all patients. Thyroid-stimulating hormone (TSH) was determined in serum on the day of the pretreatment protocol starting (T0). Serum TSH was also quantified 14 days after embryo transfer (T1). At T1, serum beta-hCG was also measured. All laboratory assays were performed at the same laboratory: TSH was measured by electrochemiluminescent assays on an automatic platform (Cobas C 702; Roche Diagnostics, Milan, Italy); antithyroid antibody assays were performed by an automated chemiluminescent method on a Liaison XL (DiaSorin, Saluggia, Italy).

### 2.4. Data Collection

For all women, we collected data regarding age, body mass index, TSH at time T0 and T1, thyroid autoantibodies, days of estradiol administration, total dose of administered estradiol, endometrial thickness, and serum beta-Hcg.

### 2.5. End Points

The primary endpoint was to evaluate the incidence of TSH above the cut-off values after the FET cycle (at time T1) in groups A and B and the trend of TSH values (at time T0 and T1) in both groups. The secondary endpoint was to evaluate the incidence of TSH above the cut-off values after the FET cycle (at time T1) in subgroups A1 and A2 and the trend of TSH values (at time T0 and T1) in both subgroups. The tertiary endpoint was to evaluate the correlation between TSH levels, total dose of estradiol administered, duration of treatment, and endometrial thickness discriminating for groups and subgroups.

### 2.6. Statistical Analysis

The continuous data are expressed as median and interquartile range (IQR). The statistical analysis was performed using JMP® version Pro 14 software for Windows (SAS Institute Inc., Cary, NC, USA). The Mann–Whitney *U* test was used to compare continuous variables between the considered patient groups. The Wilcoxon signed-rank test was used to compare TSH data at times T0 and T1 within each patient group. Pearson's chi-squared (*χ*^2^) test was used to compare qualitative data. Spearman's rank correlation coefficient (*ρ*) was used to assess the relationship between variables. For all the evaluations, *p* < 0.05 was considered statistically significant.

## 3. Results and Discussion

Patients' characteristics are reported in Table 1.

**Figure 1:**
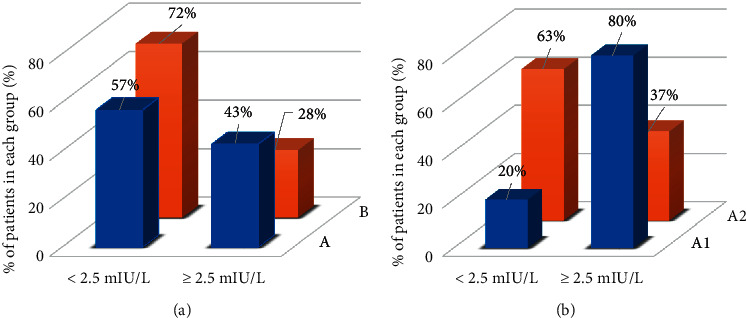
Percentage distribution of serum TSH values at T1 according to cut-off 2.5 mIU/L in groups A and B (a) and in subgroups A1 and A2 (b).

**Figure 2:**
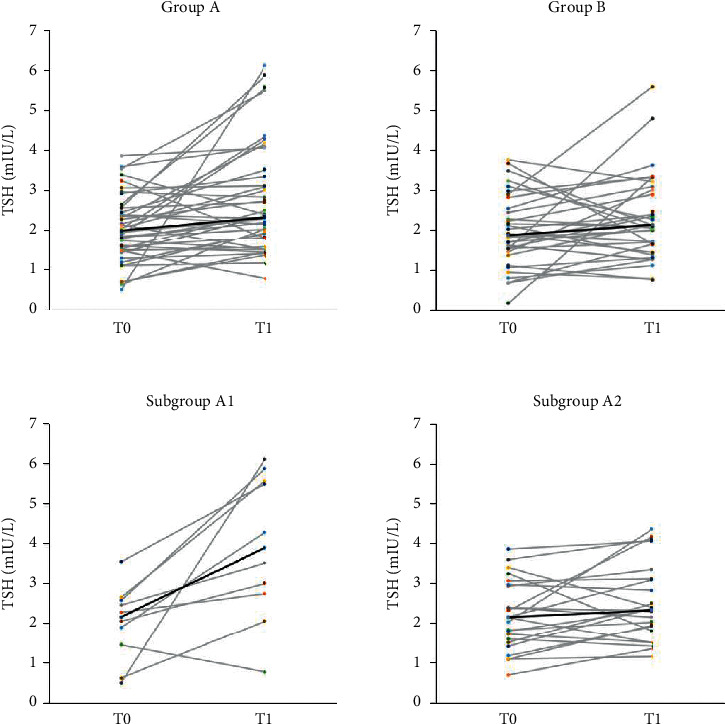
Distribution and trend of serum TSH values at T0 and T1 in groups A and B and in subgroups A1 and A2. Thick line indicates median values.

As shown in Table 1, there was no statistically significant difference in age, BMI, total estradiol dosage, and duration of treatment between groups A and B. No significant difference was found in the obtained endometrial thickness between the two groups. As well, no difference was detected between the groups for serum TSH level at T0 and T1. The incidence of TSH ≥2.5 mIU/L at T1 was 43% in group A and 28% in group B (*p*=0.17) (Figure 1(a)). In group A, a statistically significant difference was observed between serum TSH values at T0 and T1 [2.00 (IQR 1.11) mIU/L vs 2.33 (IQR 1.65) mIU/L, *p* < 0.001]. Instead, in group B, no statistically significant difference was detected between the two time points [1.87 (IQR 1.17) mIU/L vs 2.14 (IQR 1.32) mIU/L, *p*=0.13] (Figure 2).

Group A was divided into two subgroups according to the presence in serum of at least one thyroid autoantibody (TAA): 10 women (24%) were TAA positive (subgroup A1) and 24 women (57%) were TAA negative (subgroup A2), while for eight women (19%), thyroid autoimmunity was not recorded (Table 2).

Women in group A2 were statistically younger (A1: 39 (IQR 6) y; A2: 35 (IQR 7) y; *p*=0.0292). Between the two subgroups, no significant difference was found for serum TSH at T0 (group A1: 2.17 (IQR 1.35) mIU/L; A2: 2.15 (IQR 1.43) mIU/L, *p*=0.78). The incidence of values of serum TSH ≥4 mIU/L at T1 was 50% in subgroup A1 and 17% in group A2 (*p*=0.04). Instead, the incidence of TSH above 2.5 mIU/L at T1 was 80% in subgroup A1 and 37% in group A2 (*p*=0.02) (Figure 1(b)). Between subgroups A1 and A2, there was a significant difference in serum TSH levels at T1 (3.91 (IQR 3.08) mIU/L and 2.33 (IQR 1.29) mIU/L, respectively; *p*=0.0222) (Figure 2). There was no significant difference in the pregnancy rate (defined as serum *β*-hCG values >5 U/l) between groups A and B and between subgroups A1 and A2.

Values of serum TSH were not correlated with total dose of estradiol administered, duration of treatment, and endometrial thickness in any group and subgroups, except for a marginally significant value for estradiol dose and TSH at T1 in group B (*p*=0.0447). Instead, we found a significant correlation between endometrial thickness and TSH difference at T1-T0 in groups A and A2 (Tables S1 and S2).

## 4. Discussion

GnRHa is a molecule widely used in many gynecological fields and also in ARTs [5–9]. Its prolonged administration causes GnRH receptor downregulation and a subsequent hypoestrogenism state that is desired in estrogen-related diseases such as endometriosis, uterine fibroids, and breast cancer [5].

In ART fields, GnRHa has been used for many years during controlled ovarian stimulation cycles to avoid spontaneous ovulation [6]. Single GnRHa administration is used in ART for ovulation trigger when there is necessity to avoid excessive estrogen rising [14]. GnRHa is a useful option as FET pretreatment because it ensures the ability to avoid endogenous hormonal interference that could act on the endometrium and could shift the endometrial window [7–9]. However, GnRHa in depot formulation has been associated with alteration of thyroid function, both with hyper and hypothyroidism [4, 10, 11, 13]. The data about this correlation are scanty and exact pathophysiological mechanisms are not known yet and both hormonal and immune factors might be involved [11, 15–17]. Rapid changes of sex hormone globulin levels seem to play a role in thyroid function, especially when thyroid autoimmunity is present [11]. In our study, we showed that women with thyroid autoimmunity pretreated with GnRHa had a higher incidence of thyroid dysfunction. Estrogen deprivation seems not a reasonable mechanism of thyroid dysfunction because enrolled women received estradiol valerate for endometrial preparation. High estradiol levels administered could also be responsible for a decreased clearance of TGB with a subsequent greater capability of binding thyroid hormones and increased TSH [15]. Also, women in the control group received estradiol therapy.

Moreover, they received OCP as pretreatment and it did not have any significant impact on thyroid function. In the literature, it is reported that OCP can alter the fine balance between free and protein-bound thyroid hormones at the serum level by increasing or decreasing the concentration of thyroid hormone-binding proteins [18]. The usual doses of oestrogens in OCPs (20–35 *μ*g ethinyl-estradiol per day) increase serum thyroxine-binding globulin concentrations by approximately 30–50% and serum thyroxine concentrations by 20–35% [18], and these increases usually begin within two weeks [19]. However, the pharmacodynamic properties of the progestin component of OCP modify the effects of OCP itself [15]. In particular, the different progestins have different roles on thyroid function, and DNG has a bland antiestrogenic effect and an antiandrogenic action [20]. Our results are in accordance to Wiegratz I. et al.'s study in which treatment with DNG-containing OCP had only minor effects on thyroid function [21] and no variation in free thyroid hormones and TSH was revealed during the first cycle (21 days) [21]. The OCP compound (dienogest 2 mg/ethinylestradiol 0.03 mg) and the short time of use (2–3 weeks) could explain the fact that this had no significant impact on thyroid function in the patients of our study.

Other studies highlighted that GnRHa immunostimulatory actions could explain a possible mechanism involved in thyroid dysfunction. In vitro studies showed the presence of GnRH receptors in immune cells [16, 17].

**Figure 3:**
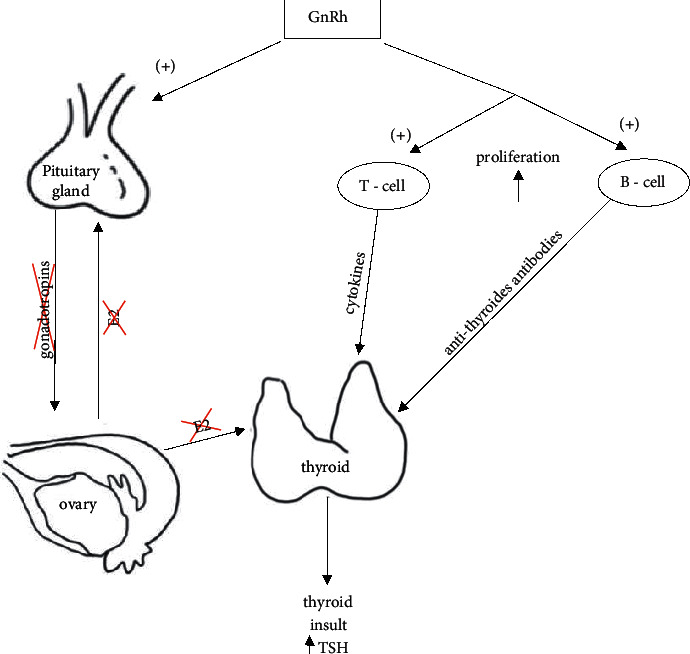
Different mechanisms of action of GnRH on thyroid. GnRH can stimulate receptors on T and B lymphocytes with release of cytokines and thyroid antibodies that act negatively on the thyroid.

Both T and B lymphocyte proliferation can be stimulated through GnRH receptors during GnRHa treatment and they can act on the thyroid through the production of cytokines and thyroid antibodies with subsequent thyroid damage and dysfunction [16] (Figure 3). In an animal model study, mice treated with GnRH antagonists had a consequent reduction in autoantibody levels [22]. In some clinical cases, the role of the GnRH agonist on immune cells has already been hypothesized [23–25]. Autoimmune thyroiditis has been reported in two case reports, respectively, after short (8 months) [23] and long (8 years) [24] initiation of the GnRHa treatment for precocious puberty. In 1997, a study reported a case of the exacerbation of lupus nephritis three weeks after GnRHa was administered for symptomatic uterine leiomyoma [25]. In our study, women pretreated with GnRHa were divided into subgroups according to the presence or absence of thyroid autoantibodies, and only women with thyroid autoimmunity reported a significant TSH alteration. Different immune cell subsets have a relevant role in the pathogenesis and tissue damage in autoimmune thyroid diseases [26, 27]. This might explain the pathophysiological mechanism of action of GnRHa on thyroid in women undergoing artificial endometrial preparation for frozen-thawed embryo transfer. Serum TSH levels measured at T1 in women pretreated with GnRHa with TAA were in the normal range in most cases; however, the American Society for Reproductive Medicine splits the TSH normal range into two subranges, < 2.5 mIU/L and >2.5 mIU/L to the upper limit [2]. TSH levels <2.5 mIU/L in infertile women undergoing ART has been associated with higher implantation rate and pregnancy rate especially when polycystic ovarian syndrome or idiopathic infertility is diagnosed [2, 28].

During the first trimester, it is recommended that serum TSH values should be lower than 2.5 mIU/L and levothyroxine should be administered when TSH is higher than 2.5 mIU/L and thyroid autoantibodies are present [1]. In the latter situation, placental alterations may occur during the first trimester with a high risk of miscarriages, premature delivery, and intrauterine growth restriction and a subsequent higher risk of neonatal injury [29, 30]. Moreover, we have previously reported that the presence of thyroid autoantibodies has a negative impact on embryo quality also in euthyroid women [31], and there is an increased miscarriage risk.

The correlation between estradiol dose and TSH at T1 in group B might be explained by an increased thyroglobulin level due to administered estradiol and subsequently fewer free thyroid hormones [15]. The correlation between endometrial thickness and TSH difference at T1-T0 in women of groups A and A2 is interesting as it is known that thyroid hormone receptors, TSH receptors, and iodothyronine deiodinase are present in human endometrium [32]. Saccardi et al. hypothesized that an increased level of TSH could stimulate endometrial TSH receptors with subsequent enhanced estrogen action [33]. A recent study underlined that the possible explanation for the improved pregnancy rate in ARTs with the GnRHa protocol could be molecular signalling at the level of endometrial receptivity [34].

## 5. Conclusions

GnRHa administration is a feasible tool for pretreatment in artificial frozen-thawed embryo transfer cycles [7, 8]. Estrogen administration seems to prevent thyroid dysfunction caused by a rapid change in estradiol levels. Even if in our study, exogenous administration of estradiol did not significantly impact thyroid function, transdermal route administration of estradiol might be recommended to avoid hepatic metabolism and TGB serum level alteration.

This study suggests that women with thyroid autoimmunity are more susceptible to thyroid dysfunction after GnRHa administration, probably due to GnRHa immunostimulatory actions, and they should be monitored closely. Probably lower basal TSH cut-off values should be reached before GnRHa administration if TAA are present.

## Figures and Tables

**Table 1:** Characteristics of the patients, according to groups A and B.

Parameter	Group A (*n* = 42)	Group B (*n* = 36)	*p* ^*∗*^
Age (y)	36 (7)	37 (4)	0.6222
BMI (kg/m^2^)	23.5 (4.5)	22.0 (2.0)	0.2732
TSH at T0 (mIU/L)	2.00 (1.11)	1.87 (1.17)	0.7185
TSH at T1 (mIU/L)	2.33 (1.65)	2.14 (1.32)	0.2066
TSH difference T1-T0 (mIU/L)	0.385 (0.99)	0.305 (1.08)	0.2357
Anti-TG Ab^†^ (n/total)	9/34	2/31	**0.0316**
Anti-TPO Ab^†^ (n/total)	8/34	2/31	0.0566
Either antithyroid Ab^†^ (n/total)	10/34	2/31	**0.0172**
Endometrial thickness (mm)	8.7 (2.0)	9.0 (1.3)	0.6574
Total dose of estradiol administered (mg)	69.0 (28.5)	71.0 (27.0)	0.8403
Duration of estradiol administration (days)	11 (4)	10 (2)	0.2071
Positive *β*-hCG test (>5 U/l) (n/total)	11/42	15/36	0.1483

Continuous variables are presented as median and (IQR). ^†^Data for some patients were not available in the clinical record. The frequency value is followed by the total number of available cases. ^*∗*^Comparison between groups A and B. Significant *p* values are indicated in italics. Patients were divided into two groups according to pretreatment protocol: 42 women received GnRHa (group A) and 36 women were given OCP (group B). None of the enrolled patients had comorbidities such as autoimmune or endocrine diseases in addition to thyroid autoimmunity.

**Table 2:** Characteristics of the patients, according to subgroups A1 and A2.

Parameter	Subgroup A1 (*n* = 10)	Subgroup A2 (*n* = 24)	*p* ^*∗*^
Age (y)	39 (6)	35 (7)	**0.0292**
BMI (kg/m^2^)	24.0 (7.25)	23.5 (3.75)	0.5406
TSH at T_0_ (mIU/L)	2.17 (1.35)	2.15 (1.43)	0.7768
TSH at T_1_ (mIU/L)	3.91 (3.08)	2.33 (1.29)	**0.0222**
TSH difference T_1_-T_0_ (mIU/L)	1.69 (2.19)	0.20 (1.00)	**0.0030**
Endometrial thickness (mm)	8.3 (1.5)	8.7 (1.9)	0.4371
Total dose of estradiol administered (mg)	65 (15)	72 (33)	0.5805
Duration of estradiol administration (days)	10.5 (4.3)	11.0 (4.0)	0.6727
Positive *β*-hCG test (>5 U/l) (*n*/total)	3/10	6/24	0.7651

Continuous variables are presented as median and (IQR). ^*∗*^Significant *p* values are indicated in italics.

## Data Availability

The research database are available on request from the first author (loris.marin@unipd.it).

## Supplementary Materials

Supplementary Materials Table S1. Pairwise correlations between the parameters considered in groups A and B. Table S2. Pairwise correlations between the parameters considered in subgroups A1 and A2.
