# Supplementary material for: Thyroid Dysfunction after Gonadotropin-Releasing Hormone Agonist Administration in Women with Thyroid Autoimmunity
Source: Int J Endocrinol. 2022 Apr 12;2022:6331657. doi: 10.1155/2022/6331657 (PMC9019438; doi:10.1155/2022/6331657)
Supplement: Supplementary Materials — Table S1. Pairwise correlations between the parameters considered in groups A and B. Table S2. Pairwise correlations between the parameters considered in subgroups A1 and A2. [file 6331657.f1.docx]

**Supplementary Table 1. Pairwise correlations (Spearman’s ρ coefficient) between the parameters considered in groups A and B.**

| Variable vs | Variable | Group A (*n*=42) | |  | Group B (*n*=36) | |
| --- | --- | --- | --- | --- | --- | --- |
|  |  | **ρ** coefficient | *p* |  | **ρ** coefficient | *p* |
| TSH at T_0_ | Estradiol dose | -0.1646 | 0.2976 |  | -0.0086 | 0.9615 |
| TSH at T_1_ | Estradiol dose | 0.0499 | 0.7537 |  | -0.3366 | ***0.0447*** |
| TSH at T_0_ | Days estradiol administration | -0.1842 | 0.2429 |  | -0.0665 | 0.7088 |
| TSH at T_1_ | Days estradiol administration | -0.0271 | 0.8647 |  | -0.3004 | 0.0750 |
| TSH at T_0_ | Endometrial thickness | 0.0886 | 0.5768 |  | 0.0692 | 0.7165 |
| TSH at T_1_ | Endometrial thickness | -0.2685 | 0.0855 |  | 0.0489 | 0.7903 |
| TSH difference T_1_-T_0_ | Estradiol dose | 0.1337 | 0.3987 |  | -0.2852 | 0.1020 |
| TSH difference T_1_-T_0_ | Days estradiol administration | 0.0732 | 0.6451 |  | -0.1886 | 0.2855 |
| TSH difference T_1_-T_0_ | Endometrial thickness | -0.3989 | ***0.0089*** |  | -0.0613 | 0.7474 |

**Supplementary Table 2. Pairwise correlations (Spearman’s ρ coefficient) between the parameters considered in subgroups A1 and A2.**

| Variable vs | Variable | Subgroup A1 (*n*=10) | |  | Subgroup A2 (*n*=24) | |
| --- | --- | --- | --- | --- | --- | --- |
|  |  | **ρ** coefficient | *p* |  | **ρ** coefficient | *p* |
| TSH at T_0_ | Estradiol dose | -0.1829 | 0.6130 |  | -0.0516 | 0.8108 |
| TSH at T_1_ | Estradiol dose | 0.2317 | 0.5195 |  | 0.1490 | 0.4872 |
| TSH at T_0_ | Days estradiol administration | -0.2018 | 0.5760 |  | -0.0315 | 0.8839 |
| TSH at T_1_ | Days estradiol administration | 0.0245 | 0.9465 |  | 0.1649 | 0.4413 |
| TSH at T_0_ | Endometrial thickness | 0.0851 | 0.8152 |  | 0.3717 | 0.0737 |
| TSH at T_1_ | Endometrial thickness | -0.3587 | 0.3088 |  | 0.0240 | 0.9114 |
| TSH difference T_1_-T_0_ | Estradiol dose | 0.3476 | 0.3251 |  | 0.1757 | 0.4116 |
| TSH difference T_1_-T_0_ | Days estradiol administration | 0.0122 | 0.9732 |  | 0.1882 | 0.3785 |
| TSH difference T_1_-T_0_ | Endometrial thickness | -0.3283 | 0.3544 |  | -0.4130 | ***0.0449*** |
